# Dorsal subscapularis approach for the surgical drainage of subscapularis intramuscular abscess: a case report

**DOI:** 10.1186/s12891-019-2852-1

**Published:** 2019-10-12

**Authors:** Ryogo Furuhata, Doji Inoue, Yasuhiro Kiyota, Hideo Morioka, Hiroshi Arino

**Affiliations:** Department of Orthopaedic Surgery, National Hospital Organization Tokyo Medical Center, 2-5-1, Higashigaoka, Meguro-ku, Tokyo, 152-8902 Japan

**Keywords:** Abscess, Subscapularis, Shoulder, Surgical drainage, Bacterial meningitis

## Abstract

**Background:**

Abscess formation in the subscapularis muscle is a rare clinical condition. Few reports are available regarding the treatment methods and surgical approaches for subscapularis intramuscular abscesses. Here, we describe a case of subscapularis intramuscular abscess that was treated successfully via surgical drainage using a new approach, the “dorsal subscapularis approach”.

**Case presentation:**

A 67-year-old woman presented to our hospital with complaints of fever and disturbance of consciousness. Two days prior to visiting our hospital, right shoulder pain and limited range of motion in the shoulder were noted. Cerebrospinal fluid examination and contrast-enhanced computed tomography (CT) imaging on admission revealed a right subscapularis intramuscular abscess with concomitant bacterial meningitis. The patient’s clinical symptoms improved after antibiotic administration for 3 weeks, but the right shoulder pain persisted. Contrast-enhanced CT imaging performed after antibiotic administration revealed an abscess in the right shoulder joint space, in addition to a capsule of the abscess in the right subscapularis muscle. We performed open surgical drainage for the abscess, which had spread from the subscapularis muscle to the glenohumeral joint. Using the deltoid-pectoral approach, we detected exudate and infected granulation tissue in the joint cavity. Furthermore, we separated the dorsal side of the subscapularis muscle from the scapula using a raspatory and detected infected granulation tissue in the subscapularis muscle belly. We performed curettage and washed as much as possible. After surgery, antibiotic administration continued for 2 weeks. The patient’s right shoulder pain subsided and CT performed 2 months after surgery revealed no recurrence of infection.

**Conclusions:**

The present case indicated that a subscapularis intramuscular abscess could lead to severe concomitant infections of other organs via the hematogenous route. Thus, early detection and treatment are necessary. Moreover, in this case, surgical drainage using a dorsal subscapularis approach was beneficial to treating the abscess, which had spread from the subscapularis muscle to the glenohumeral joint.

## Background

The subscapularis muscle is an infrequent location of abscess formation, and the diagnosis of an abscess within the subscapularis muscle is often difficult [1, 2]. Because of its rarity, few reports are available on the treatment methods for abscesses located in the subscapularis muscle. Moreover, the surgical approach to the subscapularis muscle belly remains unknown.

Here, we report a case of subscapularis intramuscular abscess with concomitant bacterial meningitis. The present case suggests that surgical drainage using a new approach, deemed the “dorsal subscapularis approach”, is beneficial for treating subscapularis intramuscular abscess.

## Case presentation

**Fig. 1:**
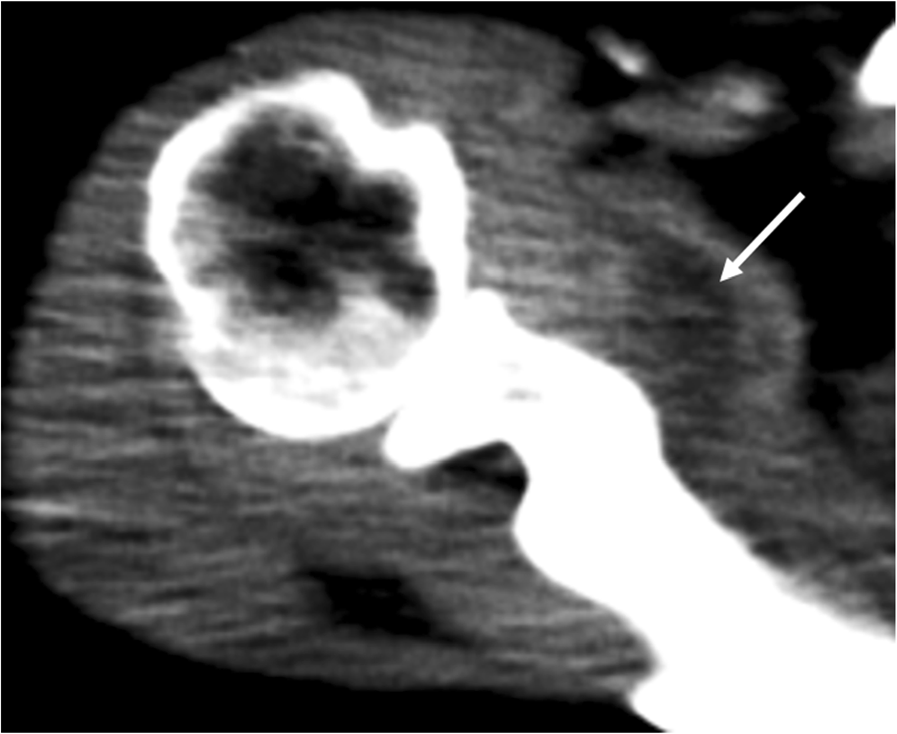
Contrast-enhanced computed tomography (CT) image performed at admission demonstrated abscess formation in the right subscapularis muscle (arrow)

**Fig. 2:**
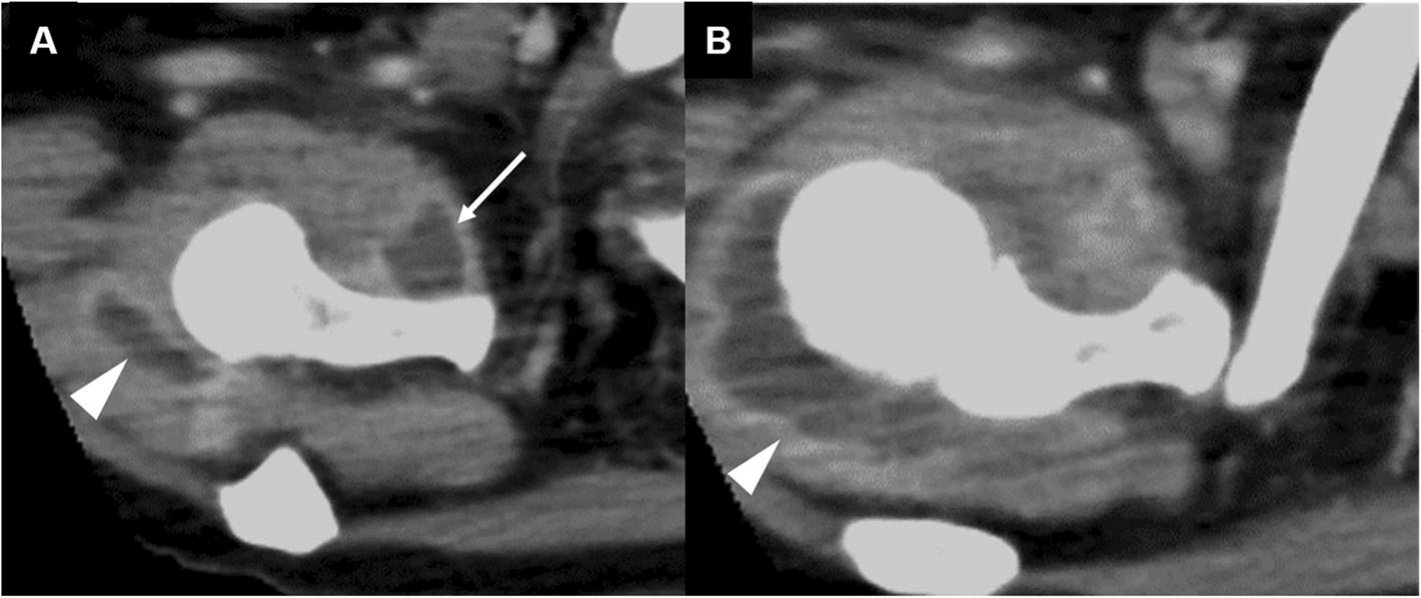
Contrast-enhanced CT imaging performed after 3 weeks of antibiotic administration revealed abscess formation in the right shoulder joint space (arrow heads), in addition to the formation of a capsule of the abscess in the right subscapularis muscle (arrow) (**a** and **b**)

**Fig. 3:**
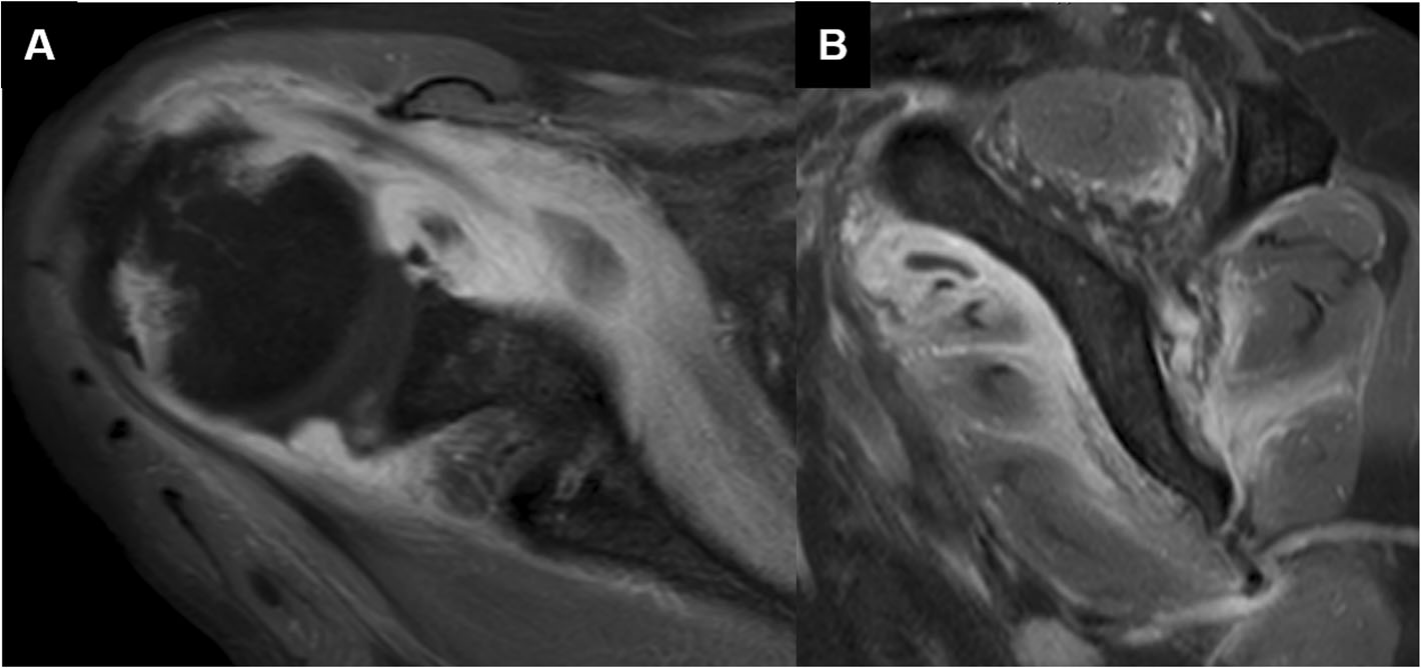
Gadolinium-enhanced fat-suppressed T1-weighted MRI oblique axial (**a**) and oblique sagittal (**b**) images revealed widespread contrast-enhanced inflammation and abscess formation within the right subscapularis muscle and glenohumeral joint space

**Fig. 4:**
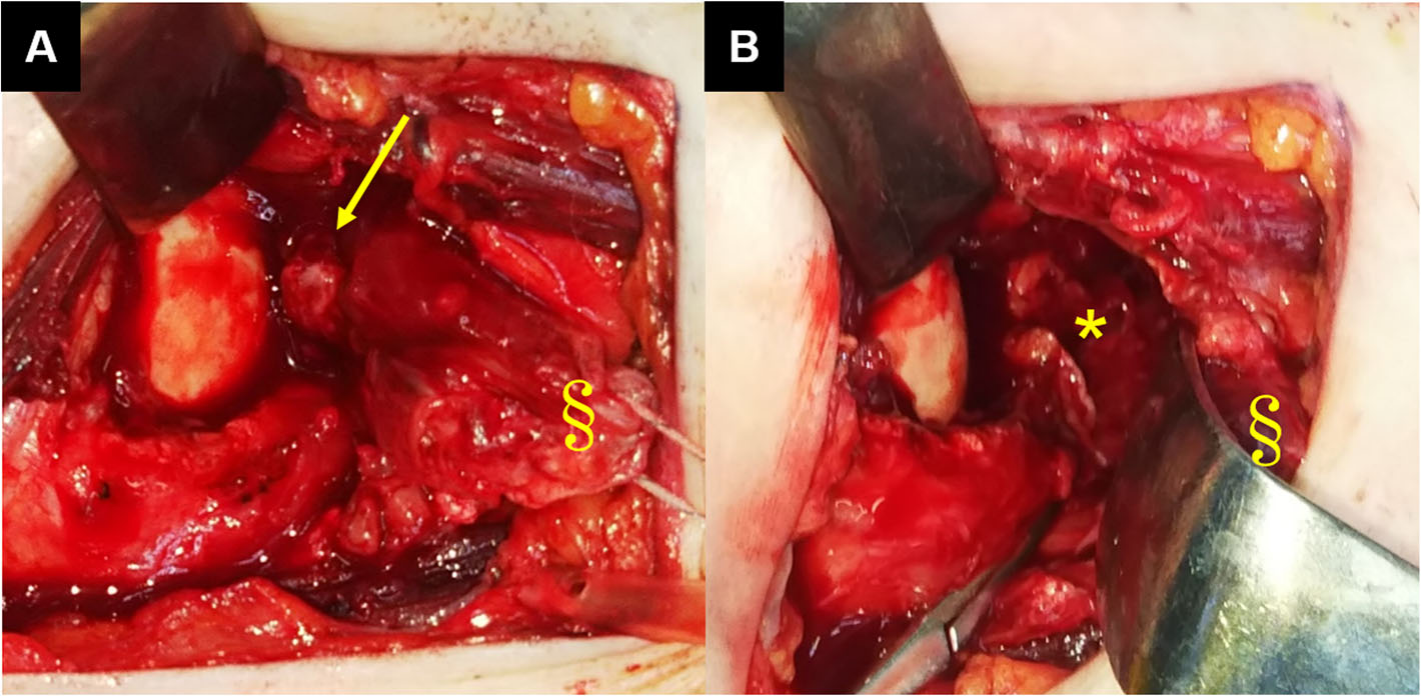
Intraoperative finding of the right shoulder. Via the deltoid-pectoral approach, we inverted the subscapularis muscle by applying FiberWire® to the subscapular tendon (section sign), and separated the subscapularis muscle from the scapula using a raspatory. We detected infected granulation tissue in the subscapularis muscle belly (arrow) (**a**). After performing curettage, the infected granulation tissue was removed under macroscopic view (asterisk) (**b**)

A 67-year-old woman presented at our hospital with complaints of fever and disturbance of consciousness. The patient had no significant past medical history. However, two days prior to visiting our hospital, fever and right shoulder pain were noted. On admission, the patient complained of headache and neck stiffness. Cerebrospinal fluid examination showed that the cell count had elevated to 3467/μL. Hematologic examination revealed a white blood cell count of 16,700/μL, a C-reactive protein (CRP) level of 29.57 mg/dL, and high inflammatory response. Bacterial meningitis was suspected and the patient was hospitalized. Ceftriaxone (4 g/day), vancomycin (1.8 g/day), and ampicillin (12 g/day) were administered for one week. Subsequently, *Streptococcus pneumoniae* was detected via blood culture, leading to a diagnosis of bacterial meningitis caused by *S. pneumoniae*. Thereafter, only ceftriaxone (4 g/day) was administered for an additional 2 weeks. Her clinical symptoms improved after 3 weeks of antibiotic administration, and the inflammatory response improved, with a 3200/μL-white blood cell count and 5.13 mg/dL-CRP level. However, the right shoulder pain and limited range of motion in the shoulder persisted. Physical examination revealed severe tenderness of the right subscapularis muscle belly and shoulder joint space, and we noted exacerbation of pain on external rotation of the shoulder joint. The active range of motion (affected side/unaffected side) elevation was 30°/150°, while internal rotation was Th8/Th3, and external rotation at the sides was 10°/60°. No limitation in passive range of motion was noted. Contrast-enhanced computed tomography (CT) performed on admission demonstrated abscess formation in the right subscapularis muscle (Fig. 1). Contrast-enhanced CT performed after 3 weeks of antibiotic administration revealed a capsule of abscess in the right subscapularis muscle, and abscess in the right shoulder joint space (Fig. 2). Gadolinium-enhanced fat-suppressed T1-weighted magnetic resonance imaging (MRI) revealed widespread contrast-enhanced inflammation within the right subscapularis muscle and glenohumeral joint space (Fig. 3). Moreover, abscess formation was observed within the inflammation (Fig. 3). These imaging findings suggested that antibiotic resistance due to abscess and capsule formation. Therefore, we planned open surgical drainage of the right subscapularis intramuscular abscess and purulent shoulder arthritis. Exposure was performed via a deltoid-pectoral approach, and a U-shaped incision was made in the subscapularis muscle. First, when the joint capsule was incised, an exudate discharged from the glenohumeral joint, and a large amount of infected granulation tissue was detected in the joint cavity. Next, we inverted the subscapularis muscle by suturing FiberWire® (Arthrex, Naples, FL, USA) to the subscapular tendon, and dissected the dorsal side of the subscapularis muscle from the scapula using a raspatory (Fig. 4a). We detected infected granulation tissue in the subscapularis muscle belly, and performed curettage as much as possible (Fig. 4b). Moreover, we obtained a favorable visual field by dissecting the short head of the biceps brachii muscle. After washing with physiological saline containing 0.3% povidone-iodine, an indwelling catheter was placed to drain the wound and the wound was closed. The dissected short head of the biceps brachii muscle was sutured to the coracoid process and the coracobrachialis muscle. After surgery, the patient’s right arm was maintained in adduction to the chest with a sling for 3 weeks and the antibiotic administration continued for 2 weeks.

**Fig. 5:**
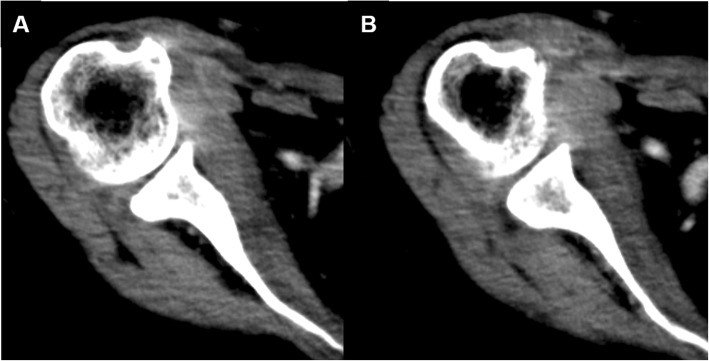
Contrast-enhanced CT imaging performed 2 months after surgery revealed no relapse of abscess (**a** and **b**)

The patient experienced no notable complications during the postoperative period. The right shoulder pain subsided and the limitation in range of motion improved. The inflammatory response improved to a 3200/μL-white blood cell count and 0.21 mg/dL-CRP level. CT performed 2 months after surgery revealed no recurrence of infection (Fig. 5).

## Discussion and conclusion

The present case indicated two clinical issues. First, a subscapularis intramuscular abscess is notably rare and its diagnosis is often difficult. However, careful attention is required since it may hematogenously lead to infection in other organs. To date, only five cases of subscapularis intramuscular abscess diagnosed using CT, MRI, or autopsy findings have been reported (Table 1) [1–5]. Immunocompromised states, such as diabetes [4] or hematoma due to shoulder trauma [3, 5], which are thought to be the sources of infection, have been reported as predisposing conditions. However, as in the present case, cases without any underlying disease have also been reported [1, 2].

**Table 1:** Summary of previous reports on abscesses occurring within the subscapularis muscle

Author	Age / Sex	Predisposing condition	Complication	Abscess region	Surgical drainage
Handorf CR (1983) [1]	19 / male	–	Sepsis Pneumonia	Subscapular space - SSc	–
Babayiğit A et al. (2009) [3]	7 / male	Blunt trauma	–	Subscapular space - SSc	–
Yilmaz G et al. (2012) [4]	9 / female	Diabetes	Sepsis Pneumonia	Subscapular space - SSc	+
Mourkus H et al. (2018) [2]	7 / male	–	–	Glenohumeral joint space - SSc	+
Patel K et al. (2018) [5]	38 / female	Blunt trauma	–	Subscapular space - SSc	+
Our case	67 / female	–	Sepsis Meningitis	Glenohumeral joint space - SSc	+

*SSc* Subscapularis

In all previous reports, the subscapularis intramuscular abscess co-occurred with abscesses involving the glenohumeral joint space or subscapular space. In the present case, contrast-enhanced CT findings suggested that the abscess that formed in the subscapularis muscle had spread to the glenohumeral joint space. Moreover, with regard to spread of infections to other organs, two cases of sepsis and pulmonary infection have been reported [1, 4], one of which was fatal. In the present case, symptoms of the right shoulder occurred first, suggesting that the subscapularis intramuscular abscess was the primary lesion that hematogenously led to the onset of bacterial meningitis. Therefore, since a subscapular muscle abscess can, via the hematogenous route, lead to severe concomitant infections of other organs, early detection and treatment are necessary.

**Fig. 6:**
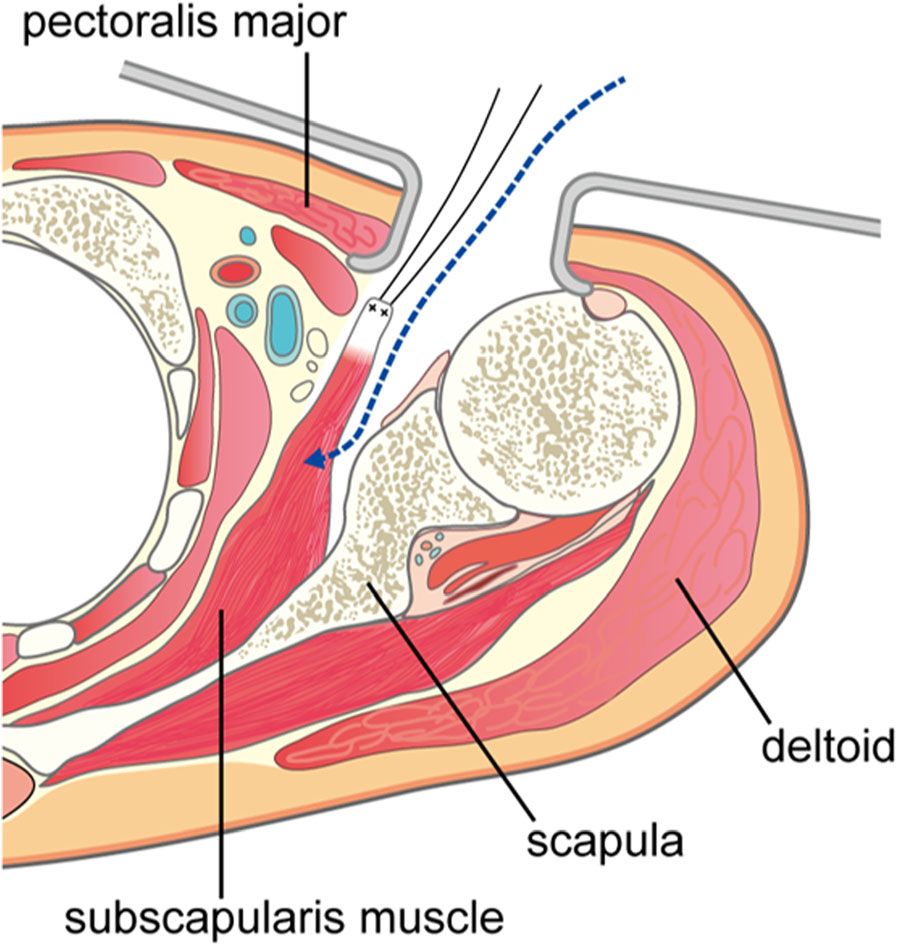
Schematic illustration for the dorsal subscapularis approach. After the deltoid-pectoral approach, we inverted the subscapularis muscle by suturing to the subscapular tendon, and dissected the dorsal side of subscapularis muscle from the scapula. This approach enable us to reach the subscapularis muscle belly with a reduced risk of vascular or nerve injury and pneumothorax

Second, the present case suggests that the dorsal subscapularis approach is useful for abscesses located in the subscapularis muscle. Treatments for abscesses in the subscapularis muscle primarily focus on antibiotics for the causative microorganism. Surgical drainage was often performed in previous reports [1, 4, 5], but few reports are available on the optimal surgical approach for this abscess type. In approaching the subscapularis muscle belly, after a deltoid-pectoral approach, both an approach from the dorsal side of the subscapularis, dissecting it from the scapula (Fig. 6), and an approach from the medial side of the coracoid process, which avoids the axillary artery, axillary vein, and nerves, were considered. In previous reports, the posterior medial approach was reported in cases with the abscess in the subscapular space [2, 6, 7]. In this approach, an incision was made medial and parallel to the medial border of the scapula, dissecting the rhomboid muscle or dividing the trapezius muscle and gaining exposure from the medial side of the scapula to the subscapular space. In the present case, an abscess was found in the glenohumeral joint space in addition to the subscapularis muscle belly. Thus, we used a deltoid-pectoral approach, reaching both the subscapularis muscle and glenohumeral joint space. In the approach from the medial side of the coracoid process, because temporary resection of the coracoid process and re-fixation by screw are necessary in order to obtain a field of view, there was concern regarding the possibility of the screw causing infection, in addition to the risk of nerve or vascular injury and pneumothorax. Therefore, we selected an approach between the subscapularis muscle and scapula. The advantage of this approach was a reduced risk of vascular or nerve injury and pneumothorax because the approach was made from the dorsal side of the subscapularis muscle. Although there was the risk of an insufficient field of view, it was possible to expeditiously perform curettage of the infected granulation tissue in the present case. Moreover, our case suggests that dissecting the short head of the biceps brachii enables a favorable field of view.

**Fig. 7:**
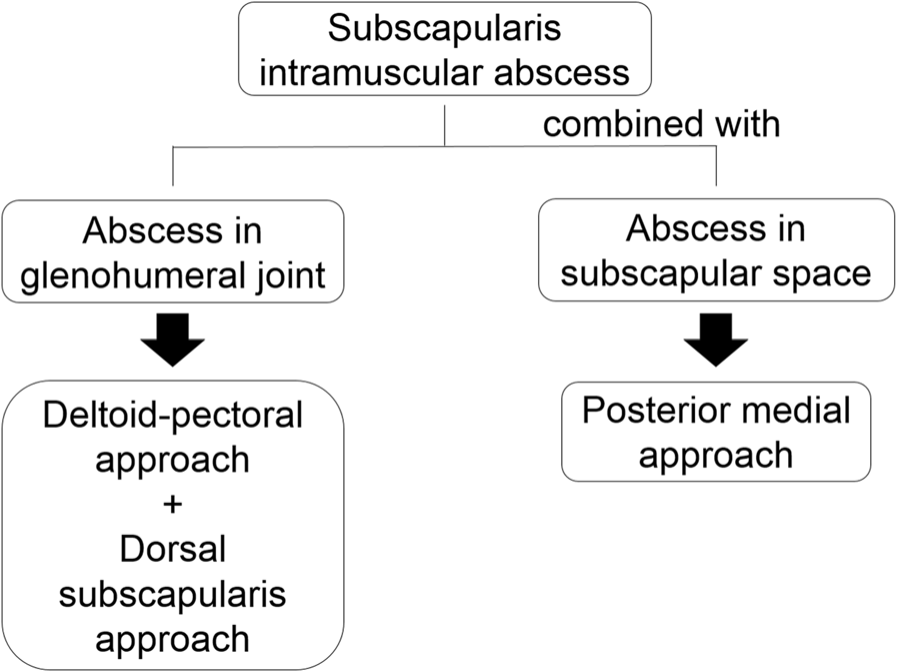
Flowchart for the surgical approach to subscapularis intramuscular abscess. This case suggests that surgical drainage using a dorsal subscapularis approach is beneficial for treating the spread of abscess from the subscapularis muscle to the glenohumeral joint. However, the posterior medial approach should be selected in cases with accompanying subscapular abscess

In summary, this paper provides new information on the surgical treatment of subscapularis intramuscular abscess (Fig. 7). The present case suggests that surgical drainage using a dorsal subscapularis approach is beneficial for treating subscapularis intramuscular abscess, especially when it co-occurs with an abscess in the glenohumeral joint. However, the posterior medial approach should be selected in cases with accompanying subscapular abscess.

## Data Availability

Data that support the findings of this study are available from the corresponding author on reasonable request.

## Abbreviations

CRP: C-reactive protein
CT: Computed tomography
MRI: Magnetic resonance imaging
